# Reconstructive Arthrodesis for Advanced Ankle and Subtalar Joint Destruction in Neuropathic and Infected Feet

**DOI:** 10.3390/jcm14134516

**Published:** 2025-06-25

**Authors:** Martin Korbel, Jaromír Šrot, Pavel Šponer

**Affiliations:** 1 Department of Orthopaedics, University Hospital Hradec Kralove, 50005 Hradec Kralove, Czech Republic; jaromir.srot@fnhk.cz (J.Š.); pavel.sponer@fnhk.cz (P.Š.); 2 Faculty of Medicine in Hradec Králové, Charles University, 50003 Hradec Kralove, Czech Republic

**Keywords:** ankle deformity, Charcot neuroarthropathy, osteomyelitis, rheumatic foot, arthrodesis, astragalectomy, amputation, neuropathy, diabetes mellitus

## Abstract

**Background/Objectives**: Advanced destruction of the ankle and subtalar joints due to neuropathy, chronic infection, or inflammatory conditions presents a major surgical challenge, often resulting in limb amputation. This descriptive retrospective study aims to evaluate outcomes of reconstructive surgery in patients, in whom limb preservation was prioritized over amputation despite significant soft tissue and osseous involvement. **Methods**: Between January 2013 and December 2022, 31 reconstructive procedures were performed on 29 patients (16 women and 13 men) with severe hindfoot deformities. Etiologies included Charcot arthropathy (55%), osteomyelitis (25%), combined pathology (10%), and rheumatoid deformity with skin defect (10%). Surgical procedures included tibiotalocalcaneal arthrodesis (39%), astragalectomy with tibiocalcaneal arthrodesis (32%), tibiotalar arthrodesis (23%), and multistage procedures (6%). Fixation methods varied based on the extent of deformity and infection. The union was assessed via radiographs and CT imaging, and outcomes were statistically analyzed using Fisher’s exact test. **Results**: Successful arthrodesis was achieved in 74% of cases (23/31). The union rate was significantly influenced by the type and level of fixation (*p* = 0.0199), with the lowest rate observed in tibiotalocalcaneal arthrodesis using external fixation (17%). Complications included surgical site infection or abscess in 42% of cases, requiring reoperation in 35%. Limb amputation was ultimately necessary in five patients (16%). **Conclusions**: Despite high complication rates, limb-preserving reconstructive surgery remains a viable alternative to amputation in selected high-risk patients with severe hindfoot pathology. Appropriate preoperative planning, tailored surgical strategy, and patient compliance are essential to achieving functional limb salvage and restoring weight-bearing capacity.

## 1. Introduction

Advanced destruction and deformity of the ankle and subtalar joints due to neuropathy, chronic infection, or inflammatory conditions remain challenging to treat and are often associated with significant morbidity [1,2,3]. Common causes include Charcot neuroarthropathy, osteomyelitis, and severe rheumatoid deformities, which progressively lead to joint instability, bone destruction, and soft tissue compromise [4,5]. The risk of complications further increases in patients with impaired vascular supply, smoking habits, or alcohol abuse [6,7,8].

Surgical treatment of advanced ankle and subtalar joint deformities with bone destruction, osteomyelitis, or soft tissue defects remains an unresolved clinical challenge. Several factors must be considered before intervention, particularly the etiology and extent of bone loss, the condition of the soft tissues, patient comorbidities, and overall compliance [9]. The goal of reconstructive surgery is not to preserve joint mobility but to achieve stable, vital bone alignment and a plantigrade, weight-bearing limb [10]. Proper reconstruction and stabilization of the ankle and subtalar joints improve foot stability, reduce skin calluses and ulceration, and enhance the quality of life [11]. Therefore, some studies recommend early arthrodesis before severe complications develop [12,13]. Ankle and subtalar fusions include isolated tibiotalar or talocalcaneal arthrodesis, tibiotalocalcaneal arthrodesis, or astragalectomy with tibiocalcaneal arthrodesis, which is indicated when the talus is destroyed or dislocated.

Amputation is generally indicated in patients with uncontrolled infection, non-salvageable bone loss, extensive soft tissue defects that cannot be reconstructed, or when previous limb salvage attempts have failed. While below-knee amputation can effectively eliminate infection and allow early mobilization, it also results in loss of proprioception and may severely limit the patient’s ability to ambulate independently, especially in neuropathic patients with balance impairment. Deciding between limb salvage and amputation requires careful evaluation of patient expectations, risk tolerance, and potential functional outcomes. In addition, many patients prefer to preserve the limb despite the risks.

The purpose of this descriptive retrospective case series was to evaluate the outcomes of reconstructive arthrodesis in patients with severe ankle and hindfoot pathology where limb preservation was prioritized over amputation, despite extensive osseous and soft tissue involvement. We hypothesized that, despite the high risk of complications, reconstruction could achieve a functional, weight-bearing limb in the majority of patients.

## 2. Materials and Methods

This retrospective study included patients who underwent reconstructive surgery for ankle and subtalar joint deformities associated with bone destruction, infection, or soft tissue defects at our department between January 2013 and December 2022.

### 2.1. Inclusion and Exclusion Criteria

Patients were included if they had bone destruction of the ankle or subtalar joint due to Charcot neuroarthropathy, osteomyelitis, or rheumatic foot deformity with a soft tissue defect. Patients with rheumatic deformities without soft tissue defects or with other etiologies were excluded. A positive preoperative culture for an infectious agent did not preclude inclusion.

All patients underwent standardized radiographic imaging (anteroposterior, lateral, and oblique views) and a CT scan to assess the extent of bone destruction and deformity. Additional laboratory tests, including inflammatory markers (CRP and ESR), microbiological cultures, and vascular status evaluation, were performed where indicated. Surgical indication and procedure planning were decided by an orthopedic surgeon specializing in foot and ankle surgery, after comprehensive review of patient comorbidities, risk factors, and compliance.

### 2.2. Surgical Timing

In Charcot neuroarthropathy, surgery was performed only after resolution of the active inflammatory phase, confirmed by clinical signs and normalized inflammatory markers. In osteomyelitis cases, definitive arthrodesis was delayed until infection stabilization, ensured by targeted antibiotic therapy guided by preoperative cultures.

### 2.3. Surgical Procedure Selection

The level of arthrodesis and fixation method were determined according to the extent and location of bone destruction, presence and site of osteomyelitis, and soft tissue condition. Tibiotalocalcaneal arthrodesis with an intramedullary nail was preferred for extensive bone loss with intact soft tissues. Isolated tibiotalar or talocalcaneal arthrodesis was performed for limited joint destruction. External fixation was chosen in patients with significant soft tissue defects or active infection. Astragalectomy was indicated for severe talus destruction or dislocation.

### 2.4. Monitored Parameters

For each patient, the following data were collected: etiology, age, sex, Body Mass Index (BMI), comorbidities, risk factors, surgical procedure (type and level of arthrodesis, fixation method, use of multistage procedures), complications, reoperations, arthrodesis union, and ability to bear weight on the operated limb and infectious status (pathogen type and culture positivity pre- and postoperatively) (Table 1).

### 2.5. Postoperative Follow-Up

Arthrodesis union was assessed both clinically (pain-free weight-bearing and absence of abnormal mobility) and radiographically (standard X-rays) at 6 and 12 weeks postoperatively, then at monthly intervals until radiographic fusion was confirmed. If fusion was uncertain, CT imaging was performed. Patients were then monitored at six-month intervals to assess long-term outcomes, with follow-up ranging from 9 months to 11 years.

### 2.6. Statistical Analysis

Data were processed and analyzed using NCSS 2023 Statistical Software (NCSS, LLC, Kaysville, UT, USA). Quantitative data are reported as median and range due to non-normal distribution. Categorical variables are presented as counts and percentages. Associations between union rates and etiology or type of fusion were tested using Fisher’s exact test in contingency tables. A *p*-value < 0.05 was considered statistically significant.

## 3. Results

The study cohort comprised 29 patients (16 women and 13 men). Two patients underwent bilateral procedures, resulting in a total of 31 surgical interventions (Table 1).

The underlying causes of deformity were Charcot arthropathy in 17 cases (55%), osteomyelitis in 8 cases (25%), a combination of Charcot arthropathy and osteomyelitis in 3 cases (10%), and rheumatic foot deformity with a soft tissue defect in 3 cases (10%). The most common comorbidity was diabetes mellitus, present in 22 patients (71%), followed by hepatopathy in 9 (29%), rheumatoid arthritis in 8 (26%), and ischemic heart disease in 4 patients (13%). Notable risk factors included active smoking in 6 patients (19%), corticosteroid therapy in 5 (16%), immunosuppression in 5 (16%), and active alcohol abuse in 4 patients (13%). The mean BMI was 29.6, with a range of 19 to 48.

Pathogenic organisms were cultured preoperatively or intraoperatively in 14 cases (45%). Staphylococcus aureus was the most frequent isolate (6 cases), followed by Enterococcus faecalis and Pseudomonas aeruginosa (2 cases each). Postoperative cultures were positive in 10 cases (32%), with Staphylococcus aureus (4 cases) and methicillin-resistant Staphylococcus aureus (MRSA) (2 cases) being the most common pathogens.

The choice of the arthrodesis level and fixation method was based on the location and extent of bone destruction, the presence of osteomyelitis, and the condition of the soft tissues. Tibiotalocalcaneal arthrodesis was the most frequent procedure, performed in 12 limbs (39%); half of these used an external fixator for stabilization and the other half an intramedullary nail. Astragalectomy combined with tibiocalcaneal arthrodesis using an external fixator was carried out in 10 limbs (32%), and tibiotalar arthrodesis was performed in 7 limbs (23%). In two cases, a multistage procedure was selected.

**Figure 1:**
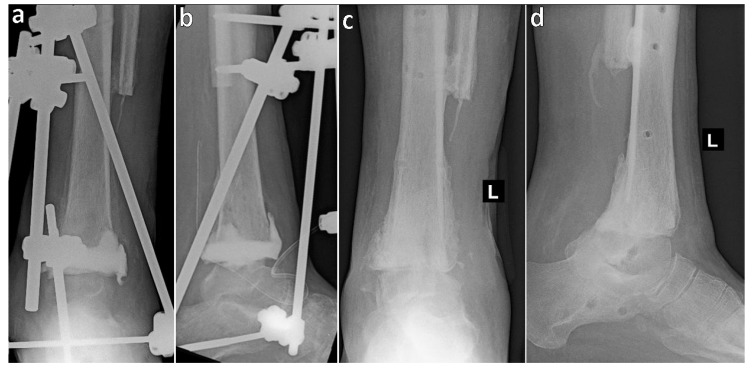
Radiographs of a patient with ankle osteomyelitis: (**a**,**b**) after resection of the tibial and talar articular surfaces with insertion of an antibiotic-loaded cement spacer and external fixation; (**c**,**d**) final status following tibiotalar arthrodesis with external fixation and autograft spongioplasty, with subsequent removal of the fixator.

**Figure 2:**
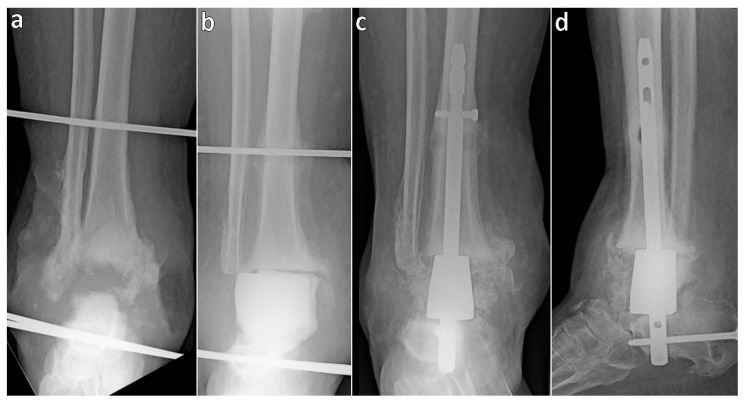
Radiographs of a patient with infected ankle nonunion: (**a**) after partial talus resection and external fixation at admission; (**b**) after complete resection and placement of a cement spacer; (**c**,**d**) final status following tibiocalcaneal arthrodesis with a trabecular metal spacer nail.

In the first multistage case, a patient with ankle osteomyelitis underwent initial resection of the articular surfaces of the tibia and talus, placement of an antibiotic-loaded cement spacer, and stabilization with an external fixator. Seven months later, the spacer was replaced with an autograft spongioplasty (Figure 1). In the second case, involving an infected ankle nonunion, the destroyed talus and tibial articular surface were resected and filled with a cement spacer, bridged with an external fixator. Two months later, the spacer was replaced with autologous bone grafts following the Masquelet technique and the soft tissue defect was covered with a rotational flap. Five months after the initial surgery, tibiocalcaneal arthrodesis with a trabecular metal spacer nail was performed (Figure 2).

Overall, successful arthrodesis was achieved in 23 of 31 cases (74%). By etiology (Table 2, Figure S1), fusion was successful in 13 of 17 Charcot arthropathy cases (76%), 7 of 8 osteomyelitis or septic arthritis cases (88%), 1 of 3 cases with combined Charcot arthropathy and osteomyelitis (33%), and 2 of 3 cases with rheumatic foot deformity and a soft tissue defect (66%). Statistical analysis showed no significant association between union rate and etiology (*p* = 0.377).

Regarding the level and type of fusion (Table 3, Figure S2), tibiotalocalcaneal arthrodesis stabilized with an external fixator had the lowest union rate, healing in only 1 of 6 cases (17%). In contrast, tibiotalocalcaneal arthrodesis with an intramedullary nail healed in 5 of 6 cases (83%). Tibiocalcaneal arthrodesis with an external fixator healed in 8 of 10 cases (80%). All tibiotalar arthrodeses and both multistage procedures achieved fusion (100%). There was a statistically significant association between the union rate and the level and type of fusion (*p* = 0.0199), primarily due to the lower success rate of tibiotalocalcaneal arthrodesis using external fixation.

Nonunion occurred in 8 interventions (26%). In three cases, the nonunion was stable and patients could bear weight with ankle-foot orthosis. However, five patients (16%) ultimately required below-knee amputation. Case 1: A patient with preoperative Staphylococcus aureus infection underwent amputation 8 months postoperatively due to progressive foot osteomyelitis. Case 2: A patient with a persistent infected nonunion of the subtalar joint required amputation 30 months after the initial procedure. Case 3: A patient with Charcot arthropathy and a soft tissue defect developed calcaneal osteomyelitis, leading to amputation 24 months after surgery. Case 4: A patient with overlap syndrome (rheumatoid arthritis and systemic lupus erythematosus) under corticosteroids and immunosuppression developed foot gangrene; despite below-knee amputation at 9 months, the patient died of sepsis. Case 5: A patient with bilateral Charcot arthropathy, alcoholism, liver cirrhosis, and chronic pancreatitis developed osteomyelitis with skin defects; an initial below-knee amputation failed to heal, resulting in transfemoral reamputation 16 months after the primary procedure. Four of these had pre-existing infections (three with Staphylococcus aureus and one with Enterococcus faecalis); all developed postoperative infections, including MRSA in two cases.

Complicated wound healing, phlegmon, or abscess formation occurred in 13 cases (42%), mostly due to pre-existing infection. In three cases, Vacuum-Assisted Closure (VAC) therapy was sufficient for healing, but ten required reoperation. An occult tibial fracture occurred in one patient during nail insertion and was subsequently treated with intramedullary fixation. In total, reoperation was necessary in 11 interventions (35%). The mean duration from primary surgery to removal of the external fixator was 117 days (range: 87–363 days).

## 4. Discussion

Reconstructive surgery for advanced deformities of the ankle and subtalar joint due to Charcot neuroarthropathy, osteomyelitis, or rheumatoid arthritis remains a significant challenge and is often associated with a high complication rate. The goal of treatment is to eliminate the infection and to achieve a limb capable of bearing weight in the plantigrade position. Although amputation may offer a definitive solution, it can severely limit independent ambulation, especially in neuropathic patients with compromised balance.

In our high-risk cohort of 31 interventions, despite significant comorbidities, the presence of infection, and soft tissue defects, the limb was preserved, and weight-bearing capacity was restored in 26 limbs. This makes our study one of the largest single-center series addressing this topic.

Previous studies have reported similarly high complication rates for ankle and subtalar arthrodesis in high-risk patients. Love et al. reported nonunion in 8 of 18 patients and wound complications in 4 cases [14]. Perlman et al. observed nonunion rates of 38% in diabetic patients and 27% in non-diabetics [15]. Other reports highlight a higher risk of reoperation when patients present with multiple risk factors such as diabetes, immunosuppression, or obesity [3,16]. In our cohort, the overall nonunion rate was 26% and infection-related complications occurred in 42% of interventions. Ultimately, five patients required amputation, highlighting the need for careful patient selection and thorough preoperative planning [17]. Patients should be informed about the considerable risk of complications, including delayed union, persistent infection, or even eventual amputation despite multiple surgical attempts. Strict compliance with postoperative care, wound hygiene, and frame management is crucial, but can be particularly demanding for neuropathic patients [11].

Charcot neuroarthropathy is characterized by progressive joint instability, deformity, soft tissue ulceration, and frequent secondary osteomyelitis [18]. Conservative management during the acute inflammatory phase includes immobilization and off-loading, but surgery is indicated once the inflammation subsides if the foot cannot be maintained in a plantigrade position.

For osteomyelitis-related deformity, the primary aim is infection control using broad-spectrum antibiotics, targeted therapy once cultures are available, and debridement of necrotic bone, followed by definitive arthrodesis once infection has resolved [19].

In our series, the choice of arthrodesis level and fixation method was tailored to the extent of bone destruction, infection, and soft tissue status. Tibiotalar arthrodesis was preferred for isolated tibiotalar joint damage, while tibiotalocalcaneal arthrodesis was used when both the tibiotalar and subtalar joints were involved or when greater mechanical stability was needed, such as in cases with severe osteoporosis. Astragalectomy with tibiocalcaneal fusion was reserved for severe talar destruction or dislocation [20,21,22]. While astragalectomy can correct deformity, it often results in limb shortening [23]. In one exceptional case in our cohort, a trabecular metal spacer nail was used to maintain leg length after talus removal.

A total of 21 interventions in our cohort were stabilized using an external fixator. This method offers important advantages, particularly in cases with soft tissue defects or active infection: by anchoring the fixator into healthy bone, it provides reliable reduction, compression at the fusion site, and stable immobilization even in compromised bone conditions [24,25,26]. Moreover, wound care is facilitated because skin defects can be dressed more easily, and adjunctive therapies such as Vacuum-Assisted Closure (VAC) can be effectively applied [27,28]. However, external fixation comes with notable drawbacks, especially the frequent occurrence of pin tract infections, which require diligent local care and can complicate healing [18,29]. In our study, this was evident in the subgroup of patients who underwent tibiotalocalcaneal arthrodesis stabilized with an external fixator—this subgroup had the lowest observed fusion rate, with successful union achieved in only 1 of 6 cases. Most patients in this group presented with a combination of Charcot neuroarthropathy, septic arthritis, and insufficient soft tissue coverage—a complex scenario that often represents a borderline indication for limb salvage. Our average duration of external fixation was 117 days (approximately 16.7 weeks). After fixator removal, we routinely applied a custom-made plastic orthosis for an additional 6 to 12 weeks to protect the fusion site during the final consolidation phase. This contrasts with other published series, where the fixator typically remains in place for 18 to 27.7 weeks without subsequent bracing [2,14,30]. We believe that the additional immobilization after frame removal may contribute to improved mechanical stability, but this approach also demands strict patient compliance and extended rehabilitation.

This study has several limitations. First, it is a retrospective descriptive case series without a control group, which limits the ability to draw direct causal inferences. Second, the sample includes a heterogeneous patient population with various comorbidities, which may affect the generalizability of the results. Third, the absence of patient-reported outcome measures and standardized functional scoring restricts the assessment of long-term functional results. Despite these limitations, the study provides valuable insight into the feasibility and challenges of limb salvage procedures in complex hindfoot pathology.

## 5. Conclusions

In conclusion, our study highlights the importance of meticulous preoperative planning, including thorough assessment of soft tissues, neurovascular status, and bone defects, to achieve successful surgical management of complex ankle and foot deformities. For patients with extensive bone destruction but intact soft tissues, we recommend tibiotalocalcaneal arthrodesis with an intramedullary nail for optimal mechanical stability. In cases with limited joint destruction, isolated tibiotalar or talocalcaneal arthrodesis is sufficient. An external fixator remains a valuable option for achieving stable arthrodesis in patients with soft tissue defects and active infection. Astragalectomy should be reserved for cases with severe talus destruction or dislocation.

Despite the high risk of complications, reconstructive surgery offers a viable alternative to amputation for selected high-risk patients, providing the potential to preserve the limb and restore weight-bearing function. Careful patient selection, individualized surgical strategy, and strict postoperative care are key to maximizing treatment success and maintaining patient mobility.

## Figures and Tables

**Table 1:** Cohort of patients included in the study.

Interv. Number	Age	Gender	BMI	Diagnosis	Risk Factors	Surgery	Reoperation	Complication	Preoperative Pathogen	Postoperative Pathogen	Union	Amp.
1	63	M	29	Charcot arthropathy with OM of the ankle and STD	DM II, RA, CHD, smoking, alcoholism	TTC arthrodesis with EF and OM debridement	below knee amputation 8Mo	purulent arthritis 4Mo, spondylodiscitis Th8/9	*Enterococcus faecalis*	*Staphylococcus aureus*	N	Y
2	61	M	48	purulent arthritis of the ankle with STD	obesity, CHD, cardiac insufficiency	TTC arthrodesis with EF			*Enterococcus faecalis*		Y	N
3	70	F	26	Charcot arthropathy with OM and ankle fracture	DM I, RA, corticotherapy	TTC arthrodesis with EF and OM debridement	rearthrodesis for infected nonunion 3Mo, below knee amputation 30Mo	purulent arthritis 3Mo, chronic OM	*Staphylococcus aureus*	*Staphylococcus aureus*	N	Y
4	34	F	34	purulent arthritis of the ankle with STD	RA, SLE, corticotherapy, immunosuppression, antiphospholipid syndrome	TTC arthrodesis with EF and lavage	below knee amputation 9Mo	stroke 5Mo, gangrene of the leg 9Mo, fatal outcome due to septic shock 9Mo	*Staphylococcus aureus*	MRSA	N	Y
5	60	M	31	Charcot arthropathy with STD	DM II, condition after pulmonary embolism	TTC arthrodesis with EF	below knee amputation 24Mo	chronic OM of the heel and ankle	*Staphylococcus aureus*	*Staphylococcus aureus*	N	Y
6	66	F	26	Charcot arthropathy with talus destruction	DM II	astragalectomy + TC arthrodesis with EF					Y	N
7	67	F	28	Charcot arthropathy with ankle fracture and STD	DM II, CHD	TT arthrodesis with EF					Y	N
8	55	F	34	Charcot arthropathy with talus destruction	DM II, nephropathy, hepatopathy	astragalectomy + TC arthrodesis with EF					Y	N
9	67	M	25	chronic OM of the ankle with fistula	DM II, hepatopathy	TT arthrodesis with EF			*Staphylococcus caprae*		Y	N
10	64	M	31	Charcot arthropathy with talus fracture	DM II, smoking	TT arthrodesis with EF	OM debridement 8Mo	OM of the ankle 8Mo		*Staphylococcus aureus*	Y	N
11	63	M	25	Charcot arthropathy with talus dislocation	DM II, smoking	astragalectomy + TC arthrodesis with EF	III. finger amputation 13Mo	III. finger gangrene 13Mo	*Pseudomonas aeruginosa*		Y	N
12	63	F	31	Charcot arthropathy	DM II, psoriatic arthritis	TTC arthrodesis with nail	VAC therapy	wound dehiscence		*Streptococcus agalactiae*	Y	N
13	71	F	31	Charcot arthropathy with talus destruction	DM II, hepatopathy, nephropathy	astragalectomy + TC arthrodesis with EF					Y	N
14	60	F	33	infected open ankle fracture with OM		(1) astragalectomy, cement spacer with gentamycin + EF (2) Masquelet autografts (3) myocutaneous flap transfer (4) TTC arthrodesis with interpositional spacer nail 5M			*Staphylococcus aureus*		Y	N
15	68	F	27	infected ankle joint nonunion with OM	LLI, CHD, hepatopathy	TT arthrodesis with EF			*Anaerococcus species*		Y	N
16	31	F	20	Charcot arthropathy	DM I, nephropathy	TT arthrodesis with screws					Y	N
17	66	F	36	Charcot arthropathy	DM II, obesity	TTC arthrodesis with nail		superficial necrosis			Y	N
18	77	F	30	Charcot arthropathy	DM II, RA	TTC arthrodesis with nail					Y	N
19	65	M	25	ankle joint nonunion with fistulized OM	smoking	TT arthrodesis with EF			*Staphylococcus aureus*		Y	N
20	49	M	37	flatfoot deformity with STD	DM II, RA, corticotherapy, hepatopathy, obesity	TTC arthrodesis with nail	stabilization of tibial fracture with ETN 4Mo	tibial fracture at the tip of the nail			Y	N
21R	65	M	25	Charcot arthropathy with talus OM and STD	chronic pancreatitis, alcoholism, smoking, secondary DM, hepatopathy	astragalectomy + TC arthrodesis with EF on the right side	debridement for abscess 4Mo	abscess of the ankle 4Mo	*Enterococcus faecalis* ESBL, *Proteus vulgaris*	*Enterococcus faecalis*	Y	N
21L	66	M	19	Charcot arthropathy with talus destruction	chronic pancreatitis, alcoholism, smoking, secondary DM, hepatopathy	astragalectomy + TC arthrodesis with EF on the left side	(1) below knee amputation 2Mo (2) tigh reamputation 16Mo	ankle OM with STD 2Mo, chronic STD 16Mo		MRSA	N	Y
23	68	F	31	infected ankle joint nonunion		TT arthrodesis with screws			*Staphylococcus aureus*		Y	N
24	71	M	37	Charcot arthropathy with talus destruction	DM II, Chronic lymphocytic leukemia, obesity	TTC arthrodesis with EF		nonunion (individual brace)			N	N
25R	75	F	30	flatfoot deformity with STD	RA, corticotherapy, immunosuppression	astragalectomy + TC arthrodesis with EF on the right side	resection of bone prominence 66Mo	STD in the planta with bone prominence 66Mo			Y	N
25L	76	F	27	flatfoot deformity with STD	RA, corticotherapy, immunosuppression	astragalectomy + TC arthrodesis with EF on the left side		nonunion (individual brace)			N	N
27	56	M	29	Charcot arthropathy	pancreas and kidney transplantation, DM I, immunosuppression	TTC arthrodesis with nail	debridement, garamycin filling, VAC therapy 3Mo	OM of the ankle 3Mo, deep vein thrombosis		*Klebsiela pneumonie*	N	N
28	80	F	24	Charcot arthropathy with talus dislocation	DM II	astragalectomy + TC arthrodesis with EF					Y	N
29	63	M	32	Charcot arthropathy with talus destruction	DM II, RA, immunosuppression	TTC arthrodesis with nail					Y	N
30	46	M	26	infected ankle joint nonunion	alcoholism, hepatopathy, tuberculosis	(1) TT resection, cement spacer with gentamycin + EF (2) TT arthrodesis with EF 7M	debridment, VAC therapy	abscess with STD 3Mo	*Staphylococcus epidermidis*	*Providencia stuarti, Pseudomonas aeruginosa*	Y	N
31	73	F	30	Charcot arthropathy with STD	DM II, hepatopathy	astragalectomy + TC arthrodesis with EF			*Pseudomonas aeruginosa*, *Streptococcus agalactiae*, *Morganela morgani*		Y	N

Abbreviations: amp. (amputation), BMI (Body Mass Index), CHD (coronary heart disease), DM (diabetes mellitus), EF (external fixator), ESBL (extended-spectrum b-lactamases), ETN (Expert Tibial Nail), F (female), interv. (intervention), L (left), LLI (lower limb ischemia), M (male), Mo (months since surgery), MRSA (methicillin-resistant Staphylococcus aureus), N (no), OM (osteomyelitis), R (right), RA (rheumatoid arthritis), SLE (systemic lupus erythematosus), STD (soft tissue defect), TC (tibiocalcaneal), TT (tibiotalar), TTC (tibiotalocalcaneal), VAC (Vacuum-Assisted Closure), Y (yes).

**Table 2:** Arthrodesis union rate in relation to etiology.

Etiology	Union	Nonunion	Union Rate
Charcot arthropathy	13	4	76%
Osteomyelitis/septic arthritis	7	1	88%
Charcot arthropathy + osteomyelitis	1	2	33%
Rheumatic foot deformity with skin defect	2	1	66%
Total	23	8	74%

**Table 3:** Arthrodesis union rate in relation to the level and type of fusion.

Location of Arthrodesis and Fixation Method	Union	Nonunion	Union Rate
tibiotalocalcaneal arthrodesis with external fixator	1	5	17%
tibiotalocalcaneal arthrodesis with nail	5	1	83%
tibiocalcaneal arthrodesis with external fixator	8	2	80%
tibiotalar arthrodesis with external fixator	5	0	100%
tibiotalar arthrodesis with screws	2	0	100%
multi-stage procedure	2	0	100%

## Data Availability

The data supporting the findings of this study are anonymized and presented to the extent necessary in Table 1 of the main text. The complete dataset is protected under personal data regulations and is available from the corresponding author upon reasonable request.

## Supplementary Materials

The following supporting information can be downloaded at: https://www.mdpi.com/article/10.3390/jcm14134516/s1, Figure S1: Arthrodesis Outcome by Etiology. Figure S2: Arthrodesis Outcome by Location and Fixation Method.

## Abbreviations

amp. (amputation), BMI (Body Mass Index), CHD (coronary heart disease), CRP (C-reactive protein), CT (computerized tomography), DM (diabetes mellitus), ESR (Erythrocyte Sedimentation Rate), EF (external fixator), ESBL (extended-spectrum b-lactamases), ETN (Expert Tibial Nail), F (female), interv. (intervention), L (left), LLI (lower limb ischemia), M (male), Mo (months since surgery), MRSA (methicillin-resistant Staphylococcus aureus), N (no), OM (osteomyelitis), R (right), RA (rheumatoid arthritis), SLE (systemic lupus erythematosus), STD (soft tissue defect), TC (tibiocalcaneal), TT (tibiotalar), TTC (tibiotalocalcaneal), VAC (Vacuum-Assisted Closure), Y (yes).

## References

[1] Johnson E.E., Weltmer J., Lian G.J., Cracchiolo A. (1992). Ilizarov ankle arthrodesis. Clin. Orthop. Relat. Res..

[2] Salem K.H., Kinzl L., Schmelz A. (2006). Ankle arthrodesis using Ilizarov ring fixators: A review of 22 cases. Foot Ankle Int..

[3] Rabinovich R.V., Haleem A.M., Rozbruch S.R. (2015). Complex ankle arthrodesis: Review of the literature. World J. Orthop..

[4] Thevendran G., Younger A., Pinney S. (2012). Current concepts review: Risk factors for nonunions in foot and ankle arthrodeses. Foot Ankle Int..

[5] Ahmad J., Raikin S.M. (2008). Ankle arthrodesis: The simple and the complex. Foot Ankle Clin..

[6] Rogers L.C., Bevilacqua N.J., Frykberg R.G., Armstrong D.G. (2007). Predictors of postoperative complications of Ilizarov external ring fixators in the foot and ankle. J. Foot Ankle Surg..

[7] Wukich D.K., Belczyk R.J., Burns P.R., Frykberg R.G. (2008). Complications encountered with circular ring fixation in persons with diabetes mellitus. Foot Ankle Int..

[8] Cobb T.K., Gabrielsen T.A., Campbell DC2nd Wallrichs S.L., Ilstrup D.M. (1994). Cigarette smoking and nonunion after ankle arthrodesis. Foot Ankle Int..

[9] Schneekloth B.J., Lowery N.J., Wukich D.K. (2016). Charcot Neuroarthropathy in Patients with Diabetes: An Updated Systematic Review of Surgical Management. J. Foot Ankle Surg..

[10] Morris H.D., Hand W.L., Dunn A.W. (1971). The modified Blair fusion for fractures of the talus. J. Bone Jt. Surg. Am..

[11] Adigweme U., Oki J.A., Johnson K., Baddaloo T., Cala M., Merrill T. Tibiocalcaneal Arthrodesis as a Limb Salvage Solution for a Patient with Rearfoot Charcot Neuroarthropathy and Avascular Necrosis of the Talus. http://www.podiatryinstitute.com.

[12] Caravaggi C., Cimmino M., Caruso S., Dalla Noce S. (2006). Intramedullary compressive nail fixation for the treatment of severe Charcot deformity of the ankle and rear foot. J. Foot Ankle Surg..

[13] Frey C., Halikus N.M., Vu-Rose T., Ebramzadeh E. (1994). A review of ankle arthrodesis: Predisposing factors to nonunion. Foot Ankle Int..

[14] Love B., Alexander B., Ray J., Halstrom J., Barranco H., Solar S., Singh M., Shah A. (2020). Outcomes of Tibiocalcaneal Arthrodesis in High-Risk Patients: An Institutional Cohort of 18 Patients. Indian J. Orthop..

[15] Perlman M.H., Thordarson D.B. (1999). Ankle fusion in a high risk population: An assessment of nonunion risk factors. Foot Ankle Int..

[16] Saxena A., DiDomenico L.A., Widtfeldt A., Adams T., Kim W. (2005). Implantable electrical bone stimulation for arthrodeses of the foot and ankle in high-risk patients: A multicenter study. J. Foot Ankle Surg..

[17] Lee D.J., Schaffer J., Chen T., Oh I. (2016). Internal Versus External Fixation of Charcot Midfoot Deformity Realignment. Orthopedics.

[18] Aikawa T., Watanabe K., Matsubara H., Nomura I., Tsuchiya H. (2016). Tibiocalcaneal Fusion for Charcot Ankle with Severe Talar Body Loss: Case Report and a Review of the Surgical Literature. J. Foot Ankle Surg..

[19] Cibura C., Lotzien S., Yilmaz E., Baecker H., Schildhauer T.A., Gessmann J. (2022). Simultaneous septic arthrodesis of the tibiotalar and subtalar joints with the Ilizarov external fixator-an analysis of 13 patients. Eur. J. Orthop. Surg. Traumatol..

[20] Reinke C., Lotzien S., Yilmaz E., Hanusrichter Y., Ull C., Baecker H., Schildhauer T.A., Geßmann J. (2022). Tibiocalcaneal arthrodesis using the Ilizarov fixator in compromised hosts: An analysis of 19 patients. Arch. Orthop. Trauma Surg..

[21] Ettinger S., Stukenborg-Colsman C., Plaass C., Yao D., Claassen L., Berger S., Waizy H., Becher C.M., Daniijidis K. (2016). Tibiocalcaneal arthrodesis as a limb salvage procedure for complex hindfoot deformities. Arch. Orthop. Trauma Surg..

[22] LaPorta G.A., Nasser E.M., Mulhern J.L. (2014). Tibiocalcaneal arthrodesis in the high-risk foot. J. Foot Ankle Surg..

[23] Kolker D., Wilson M.G. (2004). Tibiocalcaneal Arthrodesis After Total Talectomy for Treatment of Osteomyelits of the Talus. Foot Ankle Int..

[24] Yanuka M., Krasin E., Goldwirth M., Cohen Z., Otremski I. (2000). Ankle arthrodesis using the Ilizarov apparatus: Good results in 6 patients. Acta Orthop. Scand..

[25] Rochman R., Jackson Hutson J., Alade O. (2008). Tibiocalcaneal arthrodesis using the Ilizarov technique in the presence of bone loss and infection of the talus. Foot Ankle Int..

[26] Wang S., Li B., Yu X., Wu H., Liu L. (2023). Is ankle arthrodesis with an Ilizarov External Fixator an effective treatment for septic ankle arthritis? A study with a Minimum of 6 years of follow-up. Clin. Orthop. Relat. Res..

[27] Katsenis D., Bhave A., Paley D., Herzenberg J.E. (2005). Treatment of malunion and nonunion at the site of an ankle fusion with the Ilizarov apparatus. J. Bone Jt. Surg. Am..

[28] Brinkemper A., Lülsdorff R.H., Lotzien S., Kruppa C., Schildhauer T.A., Cibura C. (2024). Ilizarov fixator as salvage procedure after frustrating arthrodesis using intramedullary nailing-is there a chance of consolidation?. Arch. Orthop. Trauma Surg..

[29] Yammine K., Assi C. (2019). Intramedullary nail versus external fixator for ankle arthrodesis in Charcot neuroarthropathy: A meta-analysis of comparative studies. J. Orthop. Surg..

[30] Fragomen A.T., Borst E., Schachter L., Lyman S., Rozbruch S.R. (2012). Complex ankle arthrodesis using the Ilizarov method yields high rate of fusion. Clin. Orthop. Relat. Res..

