# Supplementary figures and images for: Reconstructive Arthrodesis for Advanced Ankle and Subtalar Joint Destruction in Neuropathic and Infected Feet
Source: J Clin Med. 2025 Jun 25;14(13):4516. doi: 10.3390/jcm14134516 (PMC12250316; doi:10.3390/jcm14134516)

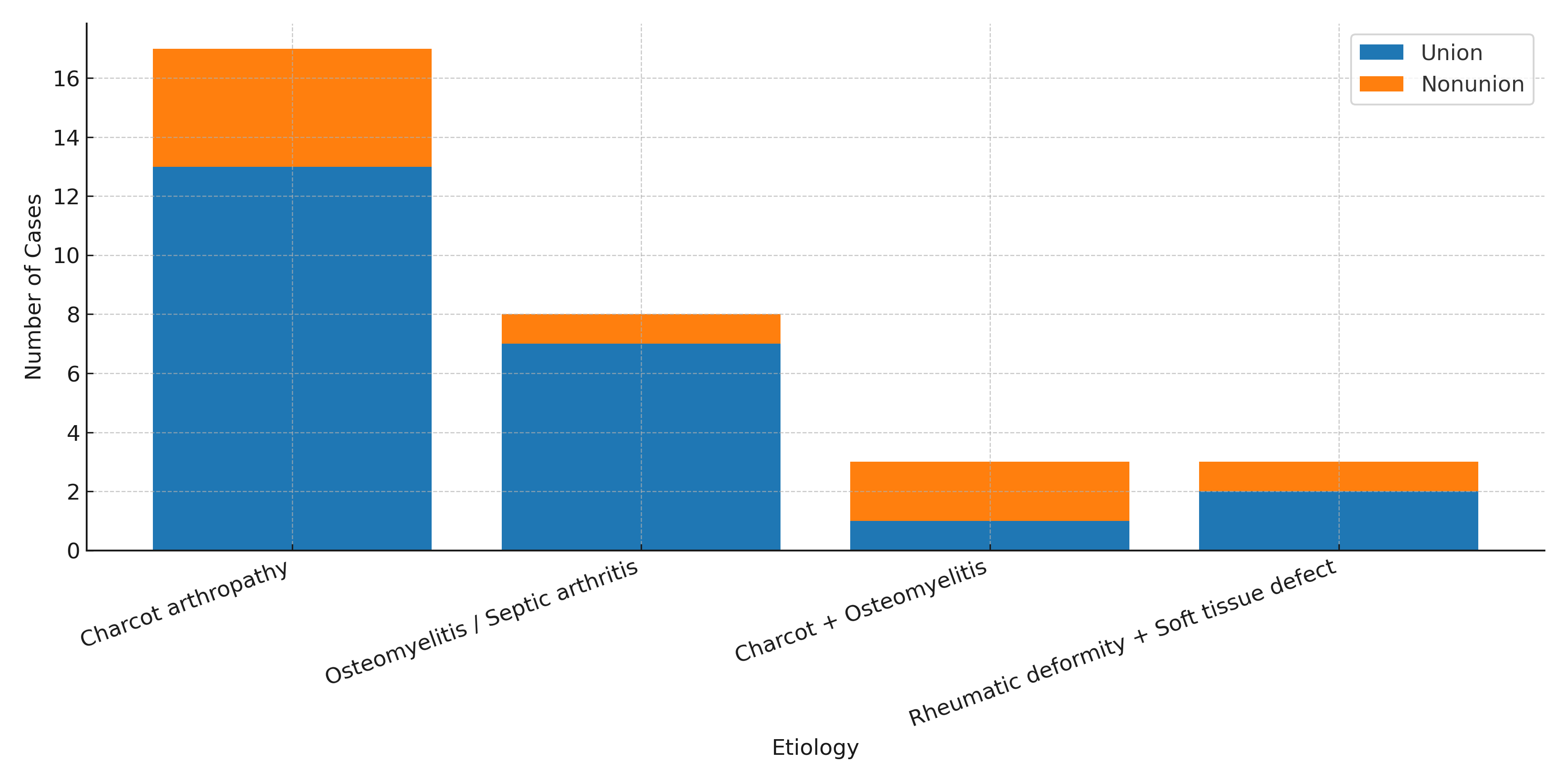

Supplement: Supplementary file 1 [file jcm-14-04516-s001.zip › Figure S1.PNG]

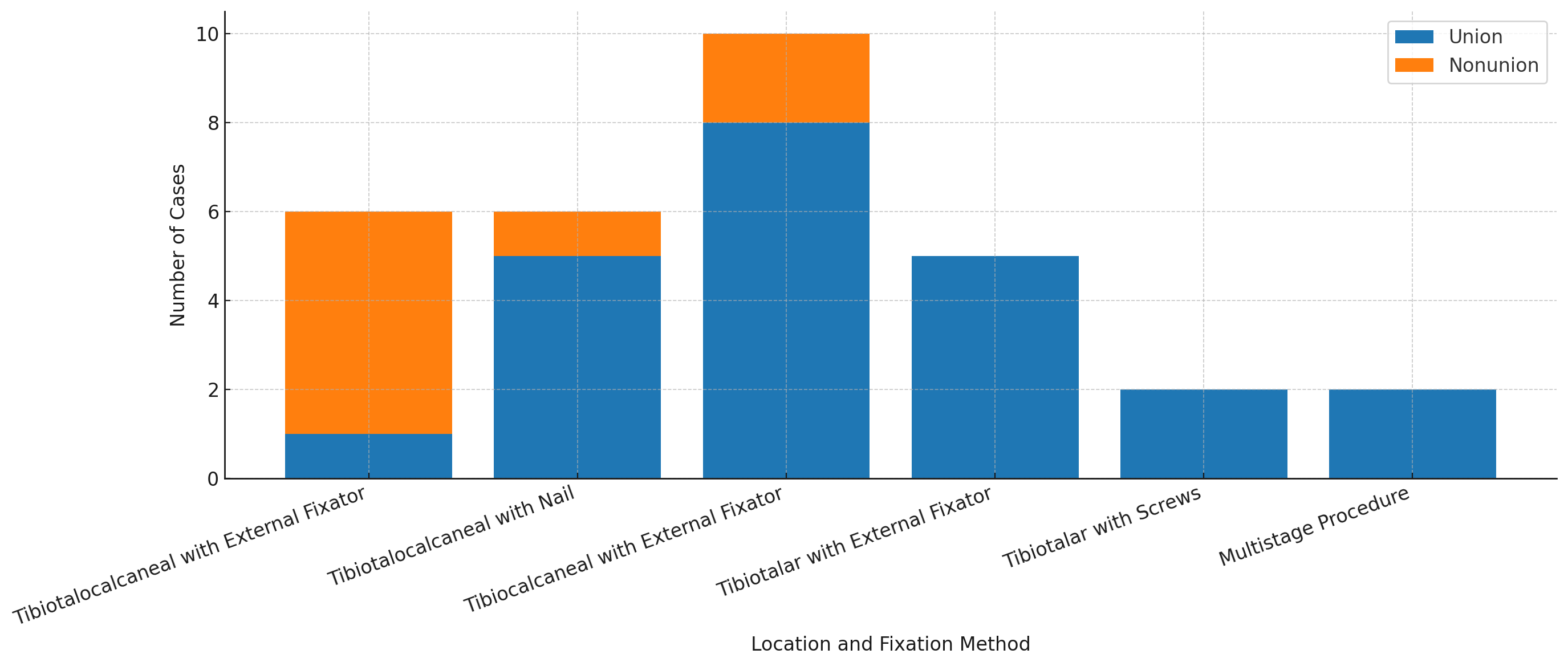

Supplement: Supplementary file 1 [file jcm-14-04516-s001.zip › Figure S2.PNG]
